# Computational discovery of *PGD*, *MAPK14*, and *KRAS* as diagnostic biomarkers for neonatal sepsis through integrated machine learning, immune infiltration analysis, and molecular docking

**DOI:** 10.3389/fimmu.2026.1808072

**Published:** 2026-05-29

**Authors:** Li Luo, Jing Chen, Weiwei Du, Jianchuan Hu

**Affiliations:** 1 Department of Neonatology Nursing, West China Second University Hospital, Sichuan University, Chengdu, China; 2 Key Laboratory of Birth Defects and Related Diseases of Women and Children, Ministry of Education, Sichuan University, Chengdu, China; 3 Sichuan Provincial Center for Mental Health, Sichuan Provincial People’s Hospital, School of Medicine, University of Electronic Science and Technology of China, Chengdu, China; 4 Department of Biotherapy, Cancer Center and State Key Laboratory of Biotherapy, West China Hospital, Sichuan University, Chengdu, China

**Keywords:** cuproptosis, diagnostic biomarkers, ferroptosis, immune infiltration, machine learning, neonatal sepsis

## Abstract

**Background:**

Neonatal sepsis is a life-threatening condition with high mortality. Ferroptosis and cuproptosis, oxidative stress-related cell death pathways, are implicated in sepsis pathogenesis, but their role in neonatal sepsis remains unclear. This study aimed to identify and validate diagnostic biomarkers for neonatal sepsis associated with ferroptosis and cuproptosis pathways using integrated bioinformatics and machine learning approaches, and to explore potential therapeutic targets.

**Methods:**

Transcriptomic data from neonatal sepsis patients (GSE69686, GSE25504) were analyzed. Differential expression analysis, weighted gene co−expression network analysis (WGCNA), and protein−protein interaction (PPI) networks were performed to identify cuproptosis− and ferroptosis−related genes (CFRGs). Three machine learning algorithms—LASSO, SVM−RFE, and XGBoost—were applied for feature selection. Immune infiltration was assessed via CIBERSORT. Molecular docking was used to screen FDA−approved drugs against candidate targets. *In vitro* validation was conducted using LPS−stimulated THP−1−derived macrophages, with gene expression measured by RT−qPCR and drug effects assessed by CCK−8 assay.

**Results:**

Three biomarkers—*PGD*, *MAPK14*, and *KRAS*—were consistently identified by all machine learning models and showed strong diagnostic performance (AUC > 0.79 in external validation). Immune infiltration analysis revealed increased neutrophils and Tregs, and decreased CD8^+^ T cells in sepsis. Molecular docking identified dasatinib and gefitinib as high−affinity binders to *MAPK14* and *KRAS*. *In vitro*, LPS stimulation significantly upregulated *PGD*, *MAPK14*, and *KRAS* expression, and candidate drugs effectively inhibited macrophage viability.

**Conclusion:**

*PGD*, *MAPK14*, and *KRAS* are promising diagnostic biomarkers for neonatal sepsis, closely linked to ferroptosis, cuproptosis, and immune dysregulation. This computational−experimental framework supports their translational potential and highlights dasatinib and gefitinib as compounds with potential anti-inflammatory activity that warrant further investigation.

## 1 Introduction

Sepsis, a life-threatening organ dysfunction caused by a dysregulated host response to infection, remains a leading cause of mortality worldwide, particularly among critically ill patients (1). A dangerous infectious disease that occurs during the newborn period is neonatal sepsis (2, 3). In low- and middle-income countries, neonatal sepsis is a major driver of preventable deaths, underscoring the urgent need for improved diagnostic and therapeutic strategies (4). Current diagnostic protocols rely heavily on time-consuming microbiological cultures, which often delay critical therapeutic interventions (5). Consequently, identifying reliable biomarkers for early diagnosis is imperative to optimize clinical outcomes.

In recent years, oxidative stress-related cell death pathways, such as ferroptosis and cuproptosis, have emerged as pivotal mechanisms in diverse pathological conditions, including cardiovascular diseases, neurodegenerative disorders, and cancer (6–9). Ferroptosis, an iron-dependent form of regulated cell death characterized by lipid peroxidation, differs mechanistically from apoptosis, necrosis, and autophagy (10). Cuproptosis, a newly identified copper-dependent cell death pathway, involves copper-induced aggregation of lipoylated mitochondrial enzymes and subsequent proteotoxic stress (11). roles of ferroptosis and cuproptosis in sepsis pathogenesis. For instance, ferroptosis contributes to sepsis-induced cardiac injury by disrupting iron homeostasis and promoting lipid peroxidation (12, 13), while cuproptosis exacerbates myocardial dysfunction through copper overload in cardiomyocytes (14). Notably, genes such as *POR*, *SLC7A5*, and *STAT3*, which regulate these pathways, have been implicated in sepsis-induced cardiomyopathy, suggesting their potential as therapeutic targets (15). Despite these advances, the specific contributions of ferroptosis- and cuproptosis-related genes to neonatal sepsis remain poorly understood, particularly their utility as diagnostic biomarkers or regulators of immune dysregulation.

This study integrates multi-omics bioinformatics and machine learning to identify CFRGs signature with diagnostic and therapeutic relevance in neonatal sepsis. Using transcriptomic data from neonatal sepsis patients and controls, we performed differential expression analysis, WGCNA, and PPI network construction to pinpoint CFRGs. Machine learning algorithms (XGBoost, LASSO, SVM-RFE) were employed to refine biomarker selection, followed by immune infiltration analysis, regulatory network mapping, and drug-target validation. Our findings unveil novel diagnostic biomarkers linked to ferroptosis and cuproptosis, offering insights into immune modulation and targeted therapy for neonatal sepsis.

## 2 Materials and methods

### 2.1 Dataset acquisition

The study retrieved clinical data and RNA sequencing data from neonatal sepsis patients from the GEO database. The GSE69686 dataset included 85 normal samples and 64 samples from patients with neonatal sepsis. The GSE25504 validation dataset included 26 neonatal sepsis samples and 37 normal samples from the GPL6947 platform and 14 neonatal sepsis samples and 6 normal samples from the GPL13667 platform (**Supplementary Table 1**, **2**). The GSE25504 dataset comprised samples from two platforms: GPL6947 (Illumina) and GPL13667 (Affymetrix). Each platform was processed independently: GPL6947 data were background-corrected and quantile-normalized using the “lumi” package, while GPL13667 data were RMA-normalized via the “affy” package. Probes were collapsed to gene symbols by retaining the probe with the highest mean expression per gene. The resulting matrices were intersected to common genes, and platform-induced batch effects were corrected using the ComBat function in the “sva” package. Removal of batch effects was confirmed by principal component analysis.

### 2.2 Identification of differentially expressed genes

DEGs between sepsis and control groups were identified using the R package “limma” package with thresholds of |log_2_FC| > 0.5 and adjusted p-value < 0.05. Volcano plots and hierarchical clustering heatmaps were generated using pheatmap.

### 2.3 Enrichment analysis

Gene Ontology (GO), Kyoto Encyclopedia of Genes and Genomes (KEGG), and Disease Ontology (DO) analyses were performed via the R package “clusterProfiler” to annotate biological processes, pathways, and disease associations of DEGs. Gene Set Enrichment Analysis (GSEA) identified hallmark pathways with significance thresholds of nominal p < 0.05 and FDR < 25%.

### 2.4 Weighted gene coexpression network analysis

Co-expression modules were constructed using the R package “WGCNA”. A soft threshold power (β = 17) ensured scale-free topology (R² > 0.85). Hierarchical clustering and dynamic tree cutting identified nine gene modules. The disease-associated module was prioritized based on module-trait correlation. Genes with module membership (MM) > 0.8 and gene significance (GS) > 0.2 were retained.

### 2.5 Construction of gene markers associated with ferroptosis and cuproptosis

Ferroptosis-related genes and cuproptosis-related genes were curated from FerrDb V2 database (http://www.zhounan.org/ferrdb/current/). PPI networks between Ferroptosis-related genes and cuproptosis-related genes were analyzed using STRING (interaction score ≥ 0.4) and visualized in Cytoscape. Overlapping genes among DEGs, WGCNA hub genes, and CFRGs were identified via Venn analysis.

### 2.6 Screening and validation of diagnostic markers

To enhance robustness and mitigate overfitting that can arise from any single method, three complementary machine learning algorithms with distinct mathematical bases were employed: LASSO (L1−regularized regression imposing sparsity), SVM−RFE (recursive feature elimination based on support vector machines), and XGBoost (gradient−boosted tree ensembles providing feature importance). The results were integrated through a consensus intersection strategy, wherein only genes selected by all three algorithms were retained as diagnostic biomarkers, as convergent selection by heterogeneous models is more likely to reflect true biological associations. LASSO regression analysis: implemented using “glmnet” package with 10-fold cross-validation to determine optimal lambda (λ). Additionally, SVM-RFE analysis: executed via the “e1071” package. XGBoost: Applied using the “xgboost” package with feature importance ranking. Common biomarkers identified by all three methods were validated in the GSE25504 cohort. Receiver operating characteristic (ROC) curves and AUC values were calculated using the “pROC” package.

### 2.7 Immune infiltration analysis

CIBERSORT quantified 22 immune cell subsets across samples. Spearman correlation analysis evaluated associations between biomarkers and immune cell fractions (p < 0.05).

### 2.8 Drug screening and molecule docking

Potential therapeutic drugs were screened using DGIdb database (https://dgidb.org/). The 3D structures of the drugs were downloaded from the PubChem database (https://pubchem.ncbi.nlm.nih.gov/). The major protein structures of the target genes were downloaded from the Protein Data Bank database (http://www.rcsb.org, PDB). Molecular docking (AutoDock Tools v1.5.7) validated drug-target interactions, with binding poses analyzed in PyMOL (v2.5.4).

### 2.9 Cell culture

THP-1 cells were cultured in RPMI-1640 medium supplemented with 10% fetal bovine serum (FBS) and maintained at 37 °C in a humidified incubator with 5% CO_2_. To differentiate THP-1 cells into M0 macrophages, cells were treated with 100 ng/mL phorbol 12-myristate 13-acetate (PMA) for 48 hours. Subsequently, M0 macrophages were polarized into the M1 phenotype by stimulation with 100 ng/mL lipopolysaccharide (LPS) and 20 ng/mL interferon−γ (IFN−γ) for an additional 48 hours.

### 2.10 Cell viability assay

Cell viability following compound treatment was assessed using the Cell Counting Kit−8 (CCK−8) assay. Briefly, THP−1−derived M1 macrophages were seeded into 96−well plates and exposed to a concentration gradient (0, 0.16, 0.31, 0.63, 1.25, 2.5, 5, 10, 20, 40, 80 μM) of each test compound for 24 hours. Thereafter, 10 μL of CCK−8 reagent was added to each well, and plates were incubated at 37 °C in the dark for the recommended duration. Absorbance was measured at 450 nm using a SpectraMax iD5 microplate reader.

### 2.11 Reverse transcription quantitative polymerase chain reaction

Total RNA was extracted using a commercial RNA extraction kit (Takara, Cat# 9767). cDNA synthesis was performed using the PrimeScript™ RT reagent kit (Takara, Cat# RR037A). Quantitative PCR was carried out using SYBR Green−based detection on a real−time PCR system. Relative gene expression was calculated via the 2−ΔΔCt method. The sequences of primers used in this study are listed below: *PGD*: Forward: 5’−TCATCGAGAAATTGAGACGGT−3’, Reverse: 5’−CACAGCAGGGTTCTCCAGTT−3’; *MAPK14*: Forward: 5’−ATGCCAAGCCATGAGGCAA−3’, Reverse: 5’−TGGGCCGCTGTAATTCTCTT−3’; *KRAS*: Forward: 5’−AGACAAGACAGAGAGTGGAGGA−3’, Reverse: 5’−AGGCATCATCAACACCCAGA−3’; *MCP−1*: Forward: 5’−CAGCCAGATGCAATCAATGCC−3’, Reverse: 5’−TGGAATCCTGAACCCACTTCT−3’; *TNF−α*: Forward: 5’−GCTGCACTTTGGAGTGATCG−3’, Reverse: 5’−GGGTTTGCTACAACATGGGC−3’; *IL−1β*: Forward: 5’−CCACAGACCTTCCAGGAGAA−3’, Reverse: 5’−GTGATCGTACAGGTGCATCG−3’; *GPX4*: Forward: 5’−CCAAGTTTGGACACCGTCTCT−3’, Reverse: 5’−TCCTTCTCTATCACCAGGGGC−3’; *ACSL4*: Forward: 5’−TTTTGCGAGCTTTCCGAGTG−3’, Reverse: 5’−AGCCGACAATAAAGTACGCAAAT−3’; *FDX1*: Forward: 5’−ACAACATATGAACCATGATAAGCTC−3’, Reverse: 5’−CCCAACCGTGATCTGTCTGT−3’.

## 3 Results

### 3.1 Screening for DEGs in neonatal sepsis

Differential expression analysis of transcriptomic data identified 480 DEGs (426 upregulated, 54 downregulated) between neonatal sepsis patients and controls (|log_2_FC| > 0.5, p < 0.05; **Supplementary Figure 1A**). Hierarchical clustering revealed distinct expression patterns for the top 25 upregulated and downregulated genes (**Supplementary Figure 1B**).

### 3.2 Functional enrichment analysis of DEGs

**Figure 1:**
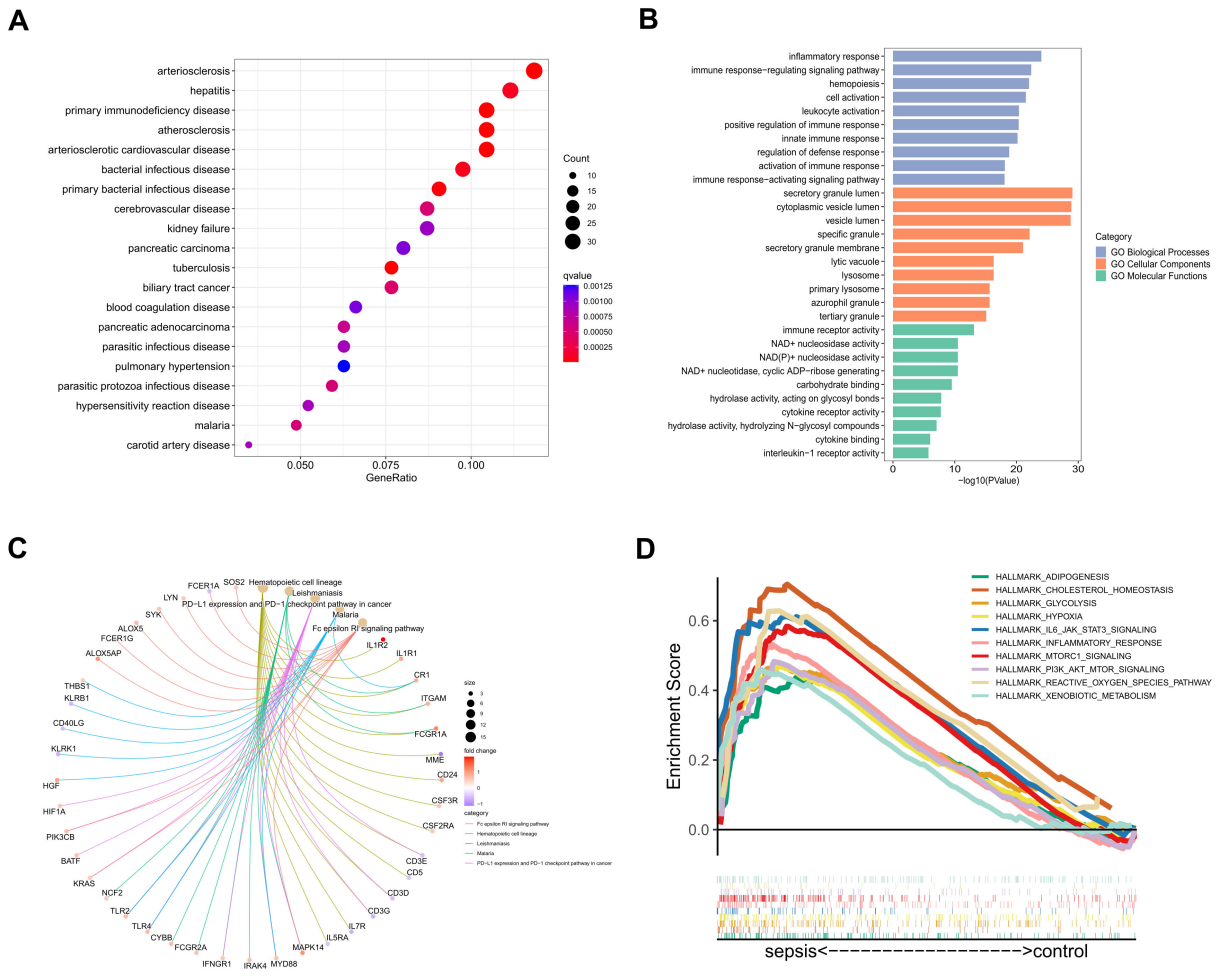
Functional analysis of DEGs. **(A)** DO analysis; **(B)** GO enrichment analysis; **(C)** KEGG pathway enrichment analysis; **(D)** GSEA.

Enrichment analyses were conducted to characterize the biological functions of DEGs. DO analysis identified significant associations with cardiovascular disorders (arteriosclerosis, atherosclerosis), infectious diseases (bacterial infections, hepatitis), and malignancies (pancreatic carcinoma) (**Figure 1A**). GO terms demonstrated predominant involvement in immune regulation (inflammatory/immune response pathways), cellular compartmentalization (secretory/vesicle lumen), and enzymatic activities (NAD(P)+ nucleosidase) (**Figure 1B**). KEGG pathway analysis highlighted enrichment in immunoreceptor signaling (Fc epsilon RI pathway), hematopoietic differentiation, and cancer-related immune regulation (PD-1/PD-L1 checkpoint) (**Figure 1C**).

GSEA of neonatal sepsis patients versus controls revealed hallmark pathway activation, including metabolic reprogramming (cholesterol homeostasis, glycolysis), stress responses (hypoxia, reactive oxygen species), and immune signaling cascades (IL6-JAK-STAT3, PI3K-AKT-mTOR) (**Figure 1D**; FDR <25%, nominal p <0.05).

### 3.3 Identification of sepsis−associated gene modules via WGCNA

**Figure 2:**
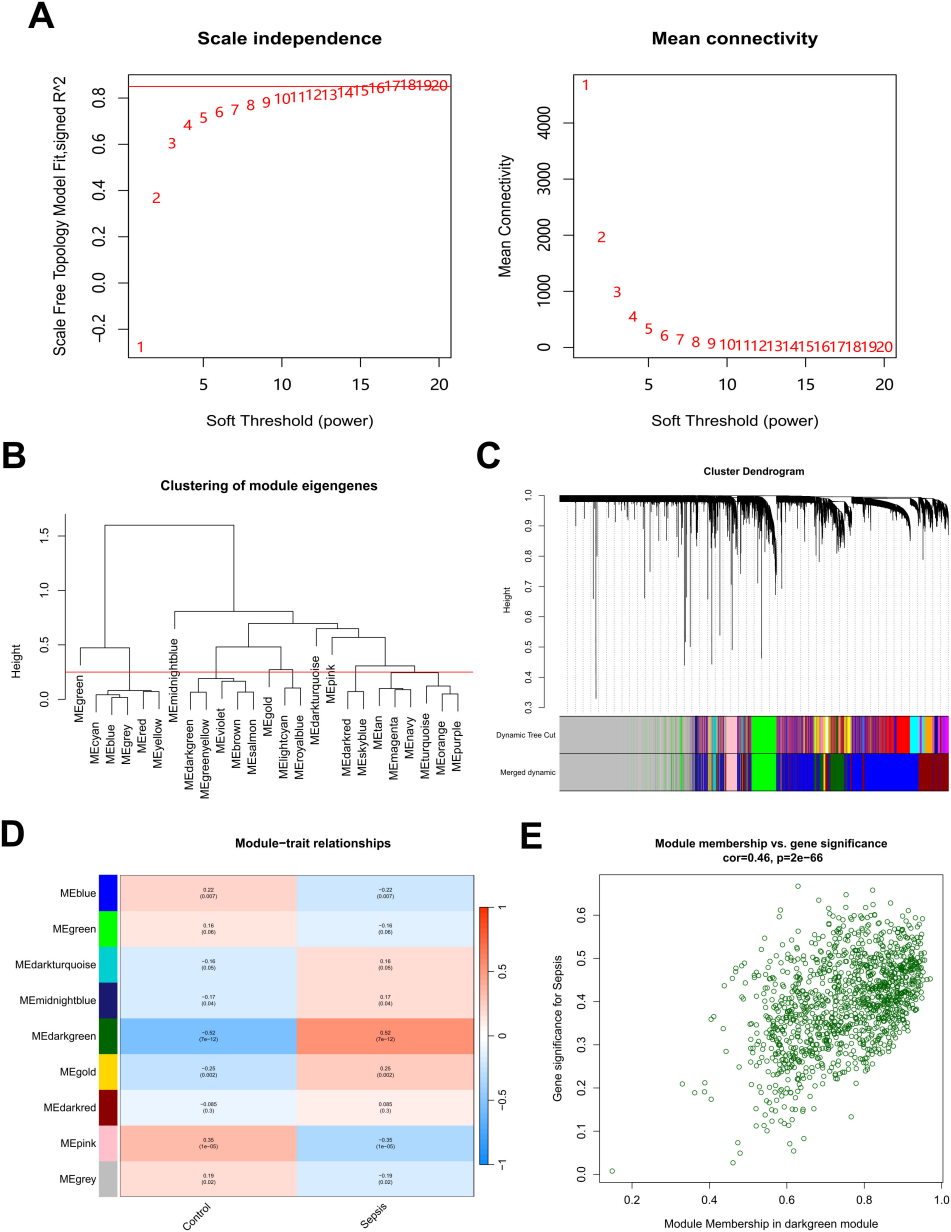
Construction of a weighted coexpression network associated with neonatal sepsis. **(A)** Scale-free exponential analysis of the soft threshold power; **(B)** Clustering dendrogram of module eigengenes; **(C)** Cluster dendrogram obtained by hierarchical clustering; **(D)** Heatmap of module-characteristic relationships; **(E)** Scatter plot of GS vs. MM for neonatal sepsis in the darkgreen module.

WGCNA was implemented to identify neonatal sepsis-associated gene modules. A soft threshold power (β = 17) ensured scale-free topology fit (R² >0.85) during network construction (**Figure 2A**). Hierarchical clustering with dynamic branch cutting delineated nine coexpression modules (**Figures 2B, C**), among which the darkgreen module exhibited the strongest clinical relevance to neonatal sepsis (Cor=0.52, p=7×10^-12^; **Figure 2D**). Gene significance (GS) and module membership (MM) displayed significant correlation in this module (Cor=0.46, p=2×10^-66^; **Figure 2E**). Applying thresholds (GS >0.2, MM >0.8), 530 module genes were prioritized as neonatal sepsis-associated candidates.

### 3.4 Machine learning for diagnostic marker screening

**Figure 3:**
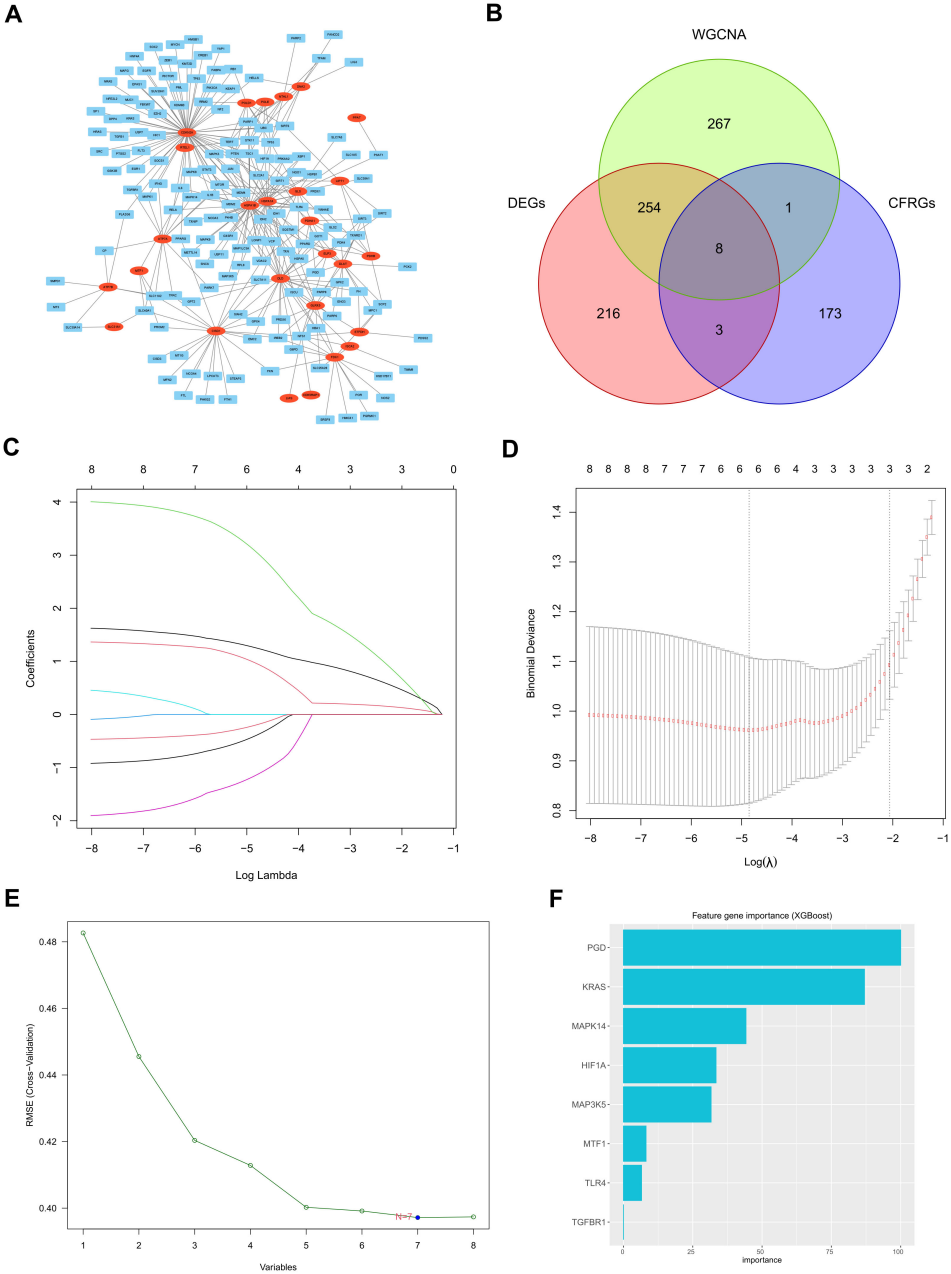
Machine learning algorithm for identifying diagnostic markers. **(A)** Correlation network of cuproptosis-related genes and ferroptosis-related genes; **(B)** Wayne plots of DEGs, WGCNA and CFRGs; **(C)** Lasso coefficient distributions of the featured genes; **(D)** Selection of the optimal LASSO model parameter (λ); **(E)** Screening of the featured genes via the SVM-RFE algorithm; **(F)** Identification of the featured genes via the XGBoost algorithm.

We retrieved 564 ferroptosis-related genes and 27 cuproptosis-related genes from FerrDb V2, constructing a PPI network using STRING database. Analysis revealed 157 ferroptosis genes exhibiting significant associations with cuproptosis genes, prompting the establishment of a combined CFRGs. Network visualization was performed using Cytoscape (**Figure 3A**). Venn analysis of DEGs, WGCNA results, and CFRGs identified eight overlapping genes (**Figure 3B**). Feature gene selection employed three machine learning approaches: LASSO regression with optimal lambda selected three key genes (**Figures 3C, D**), SVM-RFE with 10-fold cross-validation identified seven candidates (**Figure 3E**), and XGBoost analysis prioritized five genes based on importance scores (**Figure 3F**).

### 3.5 Diagnostic markers for CFRGs in neonatal sepsis patients

**Figure 4:**
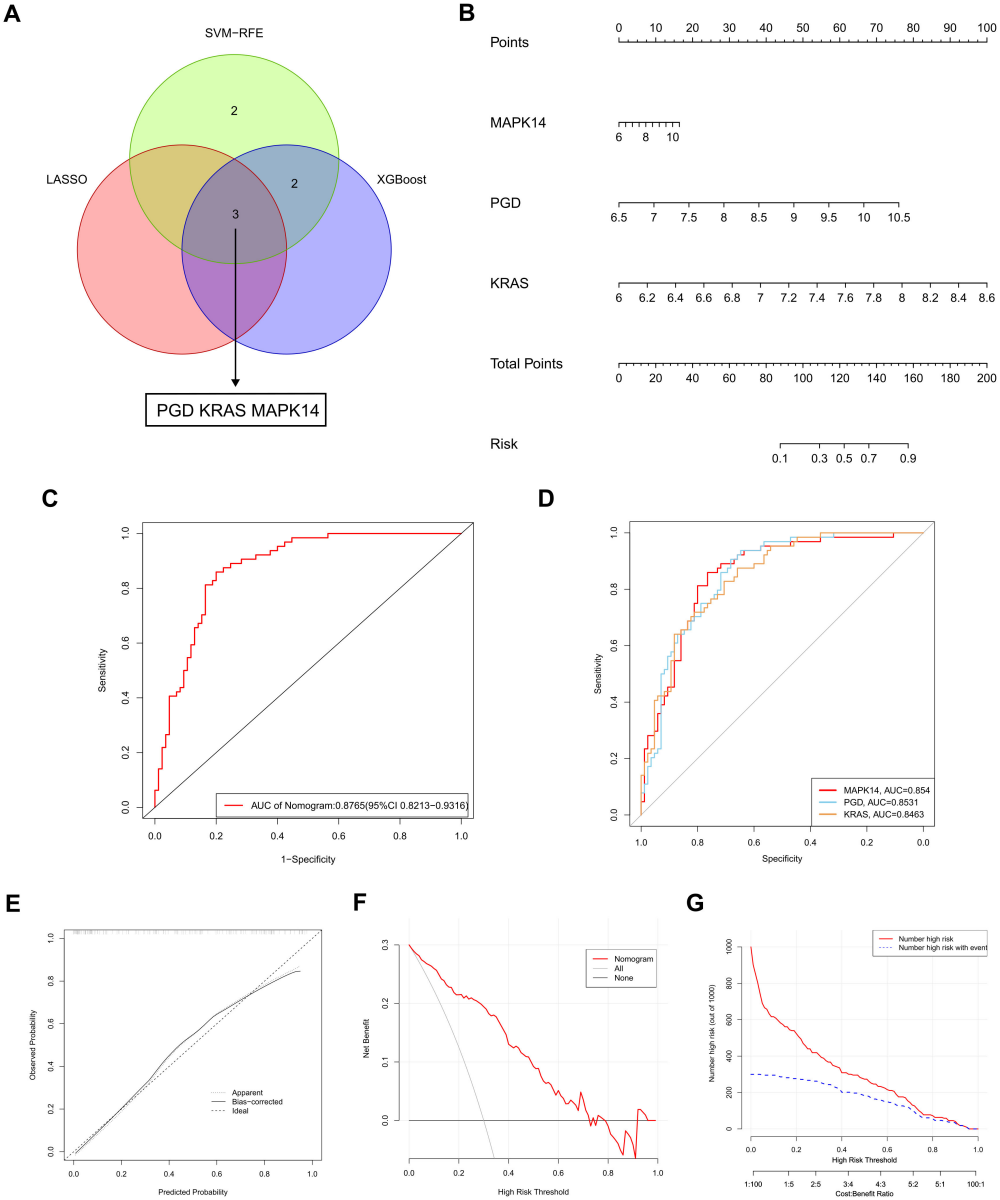
Key CFRGs genes for the diagnosis of neonatal sepsis. **(A)** Venn diagrams of diagnostic markers obtained by three machine algorithms; **(B)** Nomogram for predicting the occurrence of neonatal sepsis; **(C)** ROC curves of the nomogram; **(D)** ROC curves of the 3 diagnostic markers. **(E)** Calibration curves of the nomogram; **(F)** DCA curves; **(G)** Clinical impact curves.

Three machine learning algorithms consistently identified *PGD*, *MAPK14*, and *KRAS* as the most significant CFRGs associated with neonatal sepsis pathogenesis (**Figure 4A**). A diagnostic nomogram incorporating these three signature genes was subsequently developed to quantify sepsis risk probabilities (**Figure 4B**). ROC curve analysis revealed strong discriminative performance of the biomarkers, with AUC values of 0.853 (*PGD*), 0.854 (*MAPK14*), and 0.846 (*KRAS*) respectively (**Figures 4C, D**). Calibration curves demonstrated optimal agreement between nomogram-predicted probabilities and observed outcomes (**Figure 4E**). Decision curve analysis further confirmed the clinical utility of the model, showing superior net benefit compared to alternative diagnostic approaches across a wide threshold probability range (**Figures 4F, G**).

### 3.6 External validation of diagnostic markers

**Figure 5:**
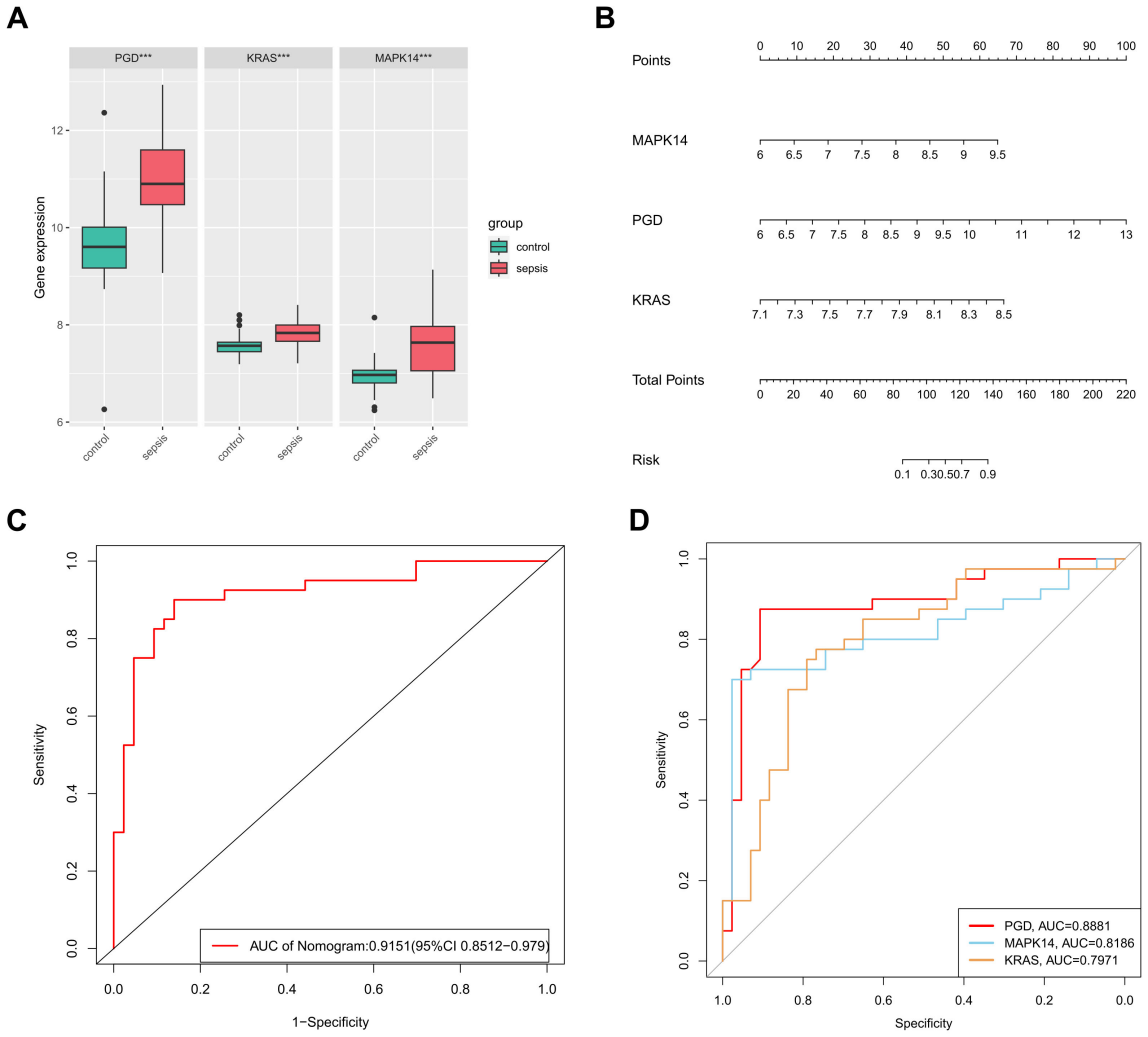
Validation of the value of diagnostic markers in the validation set. **(A)** Boxplots of the expression of key genes between the neonatal sepsis group and the control group in the validation set; **(B)** Nomogram of diagnostic markers in the validation set; **(C)** ROC curves of the nomogram in the validation set; **(D)** ROC curves of key genes in the validation set. ∗p < 0.05; ∗∗p < 0.01; ∗∗∗p < 0.001.

To independently validate the diagnostic biomarkers, validation cohorts were derived from the GSE25504 dataset (GPL13667 and GPL6947 platforms). Following the multi-platform integration and batch effect correction procedures, the two datasets were successfully merged into a harmonized validation cohort with no residual platform-driven clustering (**Supplementary Figure 2**). The sepsis cohort exhibited significantly elevated expression levels of *PGD*, *MAPK14*, and *KRAS* compared to controls (all p<0.001; **Figure 5A**). Diagnostic performance was further evaluated through a multivariable nomogram integrating these biomarkers (**Figure 5B**). ROC analysis demonstrated sustained discriminative capacity with AUC values of 0.881 (*PGD*), 0.819 (*MAPK14*), and 0.797 (*KRAS*) in external validation (**Figures 5C, D**). Notably, the significant overexpression of *PGD*, *MAPK14*, and *KRAS* in the sepsis group across both platforms (**Figure 5A**) was observable at the single−gene level prior to any multivariate modeling, and the high AUCs persisted after batch correction with no residual platform−associated clustering (**Supplementary Figure 2B**), indicating that the diagnostic performance is unlikely to be an artifact of data processing. The consistent overexpression patterns and robust classification accuracy across validation phases substantiate the reliability of these biomarkers for neonatal sepsis detection.

### 3.7 Immune infiltration landscape in neonatal sepsis

**Figure 6:**
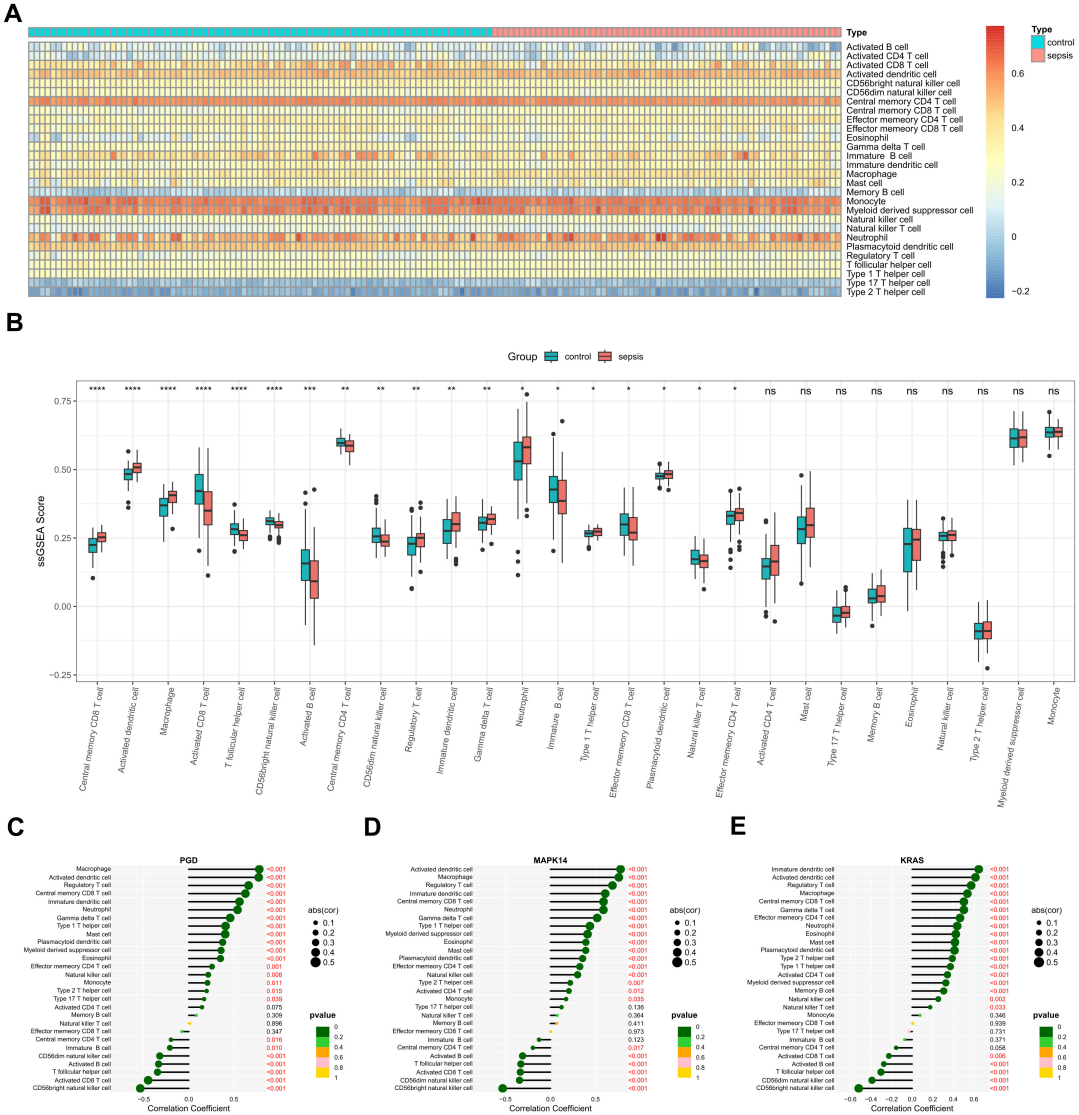
Immune infiltration in the control and neonatal sepsis groups. **(A)** Histogram of 22 infiltrating immune cells between the neonatal sepsis group and the normal group. Red name indicates normal sample, black name indicates neonatal sepsis patient sample; **(B)** Boxplot of the differences in immune cell infiltration between the neonatal sepsis group and the normal group; **(C)** Correlation of *PGD* with infiltrating immune cells; **(D)** Correlation of *MAPK14* with infiltrating immune cells; **(E)** Correlation of *KRAS* with infiltrating immune cells.

Immune cell infiltration patterns were systematically characterized in neonatal sepsis patients and healthy controls using the CIBERSORT deconvolution algorithm. Comprehensive correlation analysis revealed associations between diagnostic biomarkers and distinct immune cell subsets. The immune landscape, visualized through a stacked histogram, demonstrated marked heterogeneity in immune cell distribution across individual samples (**Figure 6A**). Comparative analysis of immune infiltration between cohorts demonstrated significant alterations in specific lymphocyte populations: sepsis patients exhibited elevated neutrophil and Treg infiltration (p<0.05), coupled with reduced proportions of CD8^+^ T cells, resting CD4^+^ memory T cells, naïve CD4^+^ T cells, and activated NK cells relative to controls (**Figure 6B**). Correlation analysis further identified statistically significant associations (p<0.05) between the diagnostic biomarkers (*PGD*, *MAPK14*, *KRAS*) and multiple immune cell subtypes, including neutrophils, Tregs, and NK cells (**Figures 6C–E**).

### 3.8 Screening potential therapeutic drugs for diagnostic biomarkers and mapping the molecular docking of potential drugs

**Figure 7:**
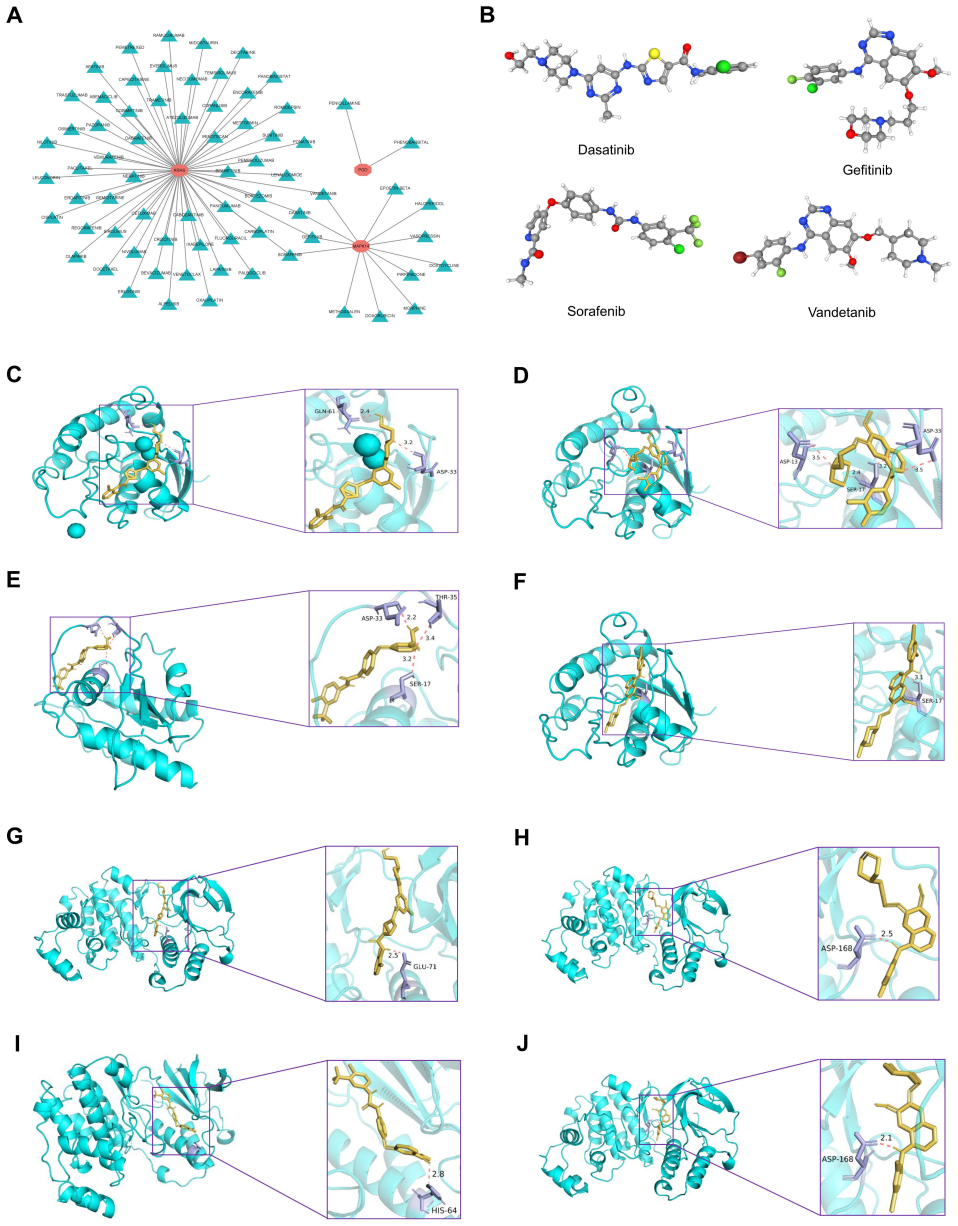
Potential drug screening for diagnostic biomarkers and molecular docking of drugs to their targets. **(A)** Potential therapeutic drugs associated with diagnostic markers; **(B)** 3D structures of four small molecule drugs; Molecular docking pattern of *KRAS* complexed with **(C)** dasatinib, **(D)** gefitinib, **(E)** sorafenib and **(F)** vandetanib; Molecular docking pattern of *MAPK14* complexed with **(G)** dasatinib, **(H)** gefitinib, **(I)** sorafenib and **(J)** vandetanib.

Potential therapeutic compounds targeting *PGD*, *MAPK14*, and *KRAS* were systematically identified through interrogation of the DGIdb database, yielding 74 FDA-approved candidates. To achieve better therapeutic outcomes, we prioritized drugs associated with at least two of these genes. However, very few drugs have been confirmed to be related to *PGD*, and none of those are associated with *MAPK14* or *KRAS*; in addition, the current literature on the role of *PGD* in sepsis is extremely limited. Therefore, we excluded *PGD* from the docking and validation analyses. Four small-molecule inhibitors—dasatinib, gefitinib, sorafenib, and vandetanib—demonstrated direct associations with *KRAS* and *MAPK14* genes (**Figure 7A**). Their three-dimensional structures were retrieved from PubChem (**Figure 7B**). Molecular docking analyses were performed using high-resolution crystal structures of MAPK14 (PDB:1A9U) and KRAS (PDB:8EBZ). AutoDock Tools 1.57 simulations revealed strong binding affinities for all compounds, with docking scores less than -7.0 kcal/mol, indicative of stable ligand-receptor interactions. Molecular docking visualizations depict critical binding conformations (**Figures 7C–J**). The small molecule compounds are represented by yellow orange, the amino acid residues by light blue, the hydrogen bonds by deep salmon dashed lines, and the hydrogen bond lengths are indicated by numbers.

### 3.9 Validation of sepsis-related gene expression and drug screening in a THP-1 cell-derived M1 macrophage model

**Figure 8:**
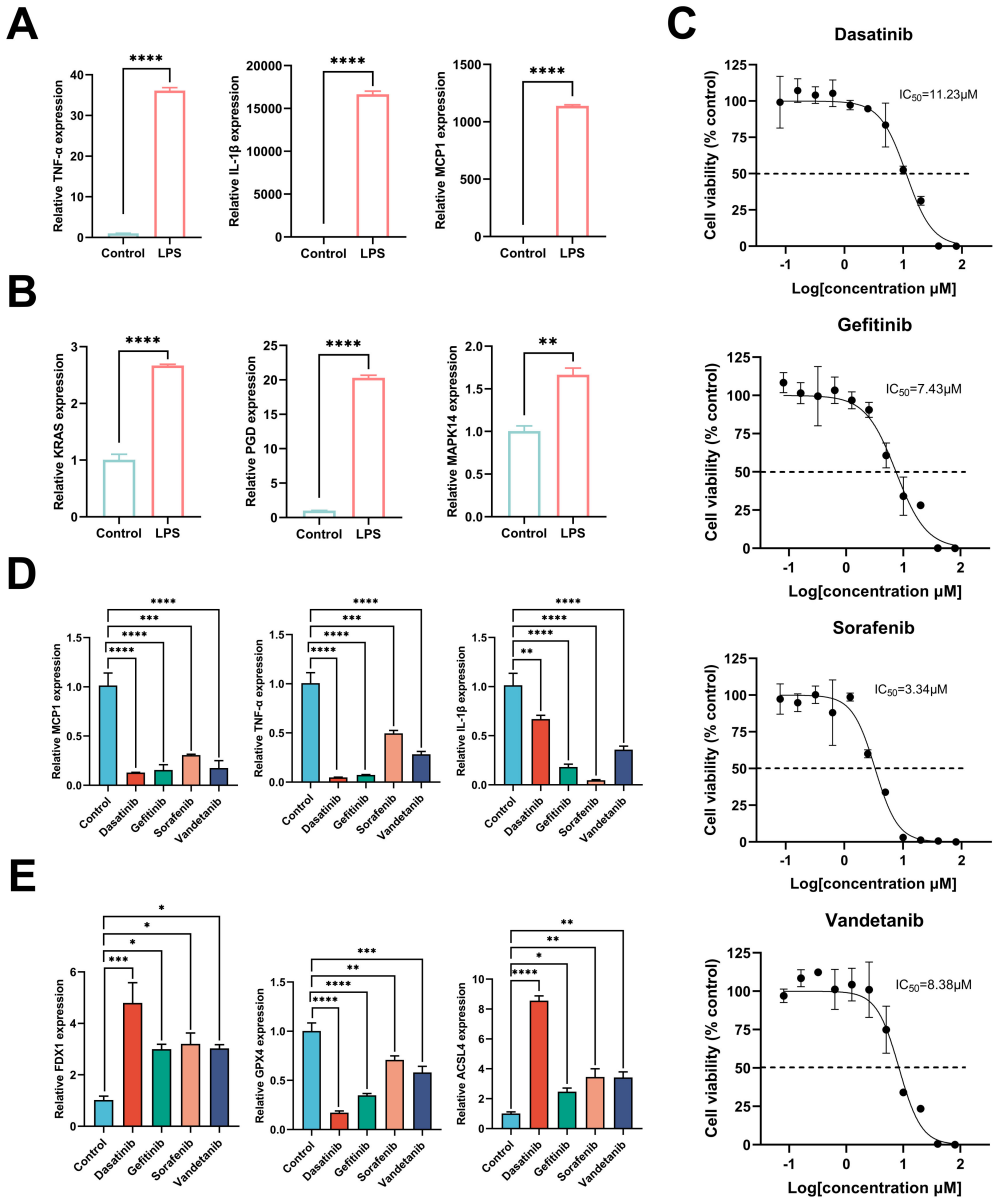
*In vitro* validation of *PGD*, *MAPK14*, and *KRAS* expression and analysis of drug inhibitory effects. **(A)** mRNA expression levels of the pro−inflammatory factors *MCP1*, *TNF−α*, and *IL−1β* in THP−1 cells and M1 macrophages. **(B)** mRNA expression levels of *PGD*, *MAPK14*, and *KRAS* in THP−1 cells and M1 macrophages. **(C)** Inhibition of THP−1−derived M1 macrophage proliferation by dasatinib, gefitinib, sorafenib, and vandetanib as assessed by CCK−8 assay. **(D)** mRNA expression levels of key pro-inflammatory cytokines (*MCP1*, *TNF-α*, *IL-1β*) following drug treatment in an LPS-stimulated M1 macrophage model. **(E)** mRNA expression levels of key cuproptosis markers (*FDX1*) and key ferroptosis markers (*GPX4*, *ACSL4*) following drug treatment in an LPS-stimulated M1 macrophage model.

To validate the expression patterns of core genes associated with sepsis, an *in vitro* inflammatory model of sepsis was first established by stimulating human THP-1 monocytic leukemia cells with LPS. Following LPS treatment, the expression levels of pro−inflammatory factors *MCP1*, *TNF−α*, and *IL−1β* were significantly upregulated in THP−1 cells, indicating successful polarization into M1−type macrophages and confirming the validity of the model (**Figure 8A**). Subsequently, qPCR analysis revealed that the expression of *PGD*, *MAPK14*, and *KRAS* was markedly increased in the LPS−induced sepsis model compared with the control group, which was consistent with predictions from prior bioinformatics analysis (**Figure 8B**).

Furthermore, based on this *in vitro* model, the inhibitory effects of four tyrosine kinase inhibitors—dasatinib, gefitinib, sorafenib, and vandetanib—on sepsis−related cellular activity were evaluated. CCK−8 assay results demonstrated that all four compounds inhibited cell viability to varying degrees, with half−maximal inhibitory concentration (IC_50_) values of 11.23 μM for dasatinib, 7.43 μM for gefitinib, 3.34 μM for sorafenib, and 8.38 μM for vandetanib (**Figure 8C**). In the LPS-stimulated M1 macrophage model, treatment with the four drugs resulted in a significant decrease in the expression levels of key pro-inflammatory factors *MCP1*, *TNF-α*, and *IL-1β*, indicating that these drugs possess anti-inflammatory effects (**Figure 8D**). The expression levels of *GPX4*, a key marker of ferroptosis, decreased significantly, whilst those of *ACSL4* increased significantly; similarly, the expression levels of *FDX1*, a key marker of cupraptosis, increased significantly. This indicates that the ferroptosis and cupraptosis pathways are indeed activated at the transcriptional level in the LPS-stimulated M1 macrophage model (**Figure 8E**).

## 4 Discussion

Neonatal sepsis remains a serious global health challenge due to its complex pathophysiology and diagnostic delays, resulting in high morbidity and mortality (16, 17). By integrating multi-omics bioinformatics with machine learning approaches, this study successfully identified and validated *PGD*, *MAPK14*, and *KRAS* as novel diagnostic biomarkers linked to ferroptosis and cuproptosis. Our findings demonstrate that these three markers not only exhibit strong diagnostic performance (external validation AUC > 0.79) but, more importantly, are closely connected to immune dysregulation and key regulated cell death pathways, providing new perspectives for early diagnosis and targeted therapy.

Given the growing evidence that both ferroptosis and cuproptosis contribute to sepsis−induced organ injury, we deliberately integrated these two gene sets into a combined CFRG panel. The rationale for this integration is threefold. First, although these pathways are mechanistically distinct, they share a common background of mitochondrial dysfunction, reactive oxygen species generation, and disruption of metal ion homeostasis—processes that are profoundly dysregulated in sepsis and often co−occur in septic tissues. Second, a single cell death pathway may not fully capture the complex oxidative damage in neonatal sepsis; combining ferroptosis− and cuproptosis−related genes provides a more comprehensive transcriptomic representation of the oxidative stress−associated cell death landscape. Third, an analogous strategy of jointly analyzing ferroptosis and cuproptosis gene signatures has been successfully used to characterize sepsis−induced cardiomyopathy (15, 18), supporting the validity of this integrated approach. The co−expression network and PPI analysis further revealed that 157 ferroptosis genes exhibit significant associations with cuproptosis genes, suggesting functional crosstalk that warranted their joint consideration.

Our results align with and extend existing research. *MAPK14* is a key regulator of inflammatory signaling and has been associated with disease severity and cardiac dysfunction in pediatric sepsis (19). Crucially, *MAPK14* has been demonstrated to directly modulate ferroptosis execution by phosphorylating *ACSL4* and regulating *GPX4* expression (20–22), establishing a direct mechanistic link between this kinase and ferroptotic cell death in the inflamed microenvironment. Activation of *KRAS* can drive pro-inflammatory pathways and mitochondrial stress, thereby exacerbating systemic inflammation (23–25). Mechanistically, oncogenic or overexpressed *KRAS* is known to upregulate transferrin receptor 1 (*TFR1*), increase intracellular iron levels, and sensitize cells to ferroptosis; moreover, *KRAS*−driven alterations in mitochondrial metabolism may influence susceptibility to cuproptosis, which relies on the lipoylation of mitochondrial enzymes (26). Although experimental studies directly investigating the role of *PGD* in sepsis remain limited, its mechanisms have been relatively well characterized in cancer and metabolic diseases. *PGD* encodes phosphogluconate dehydrogenase, a key enzyme of the pentose phosphate pathway that generates NADPH, the essential cofactor for the glutathione and thioredoxin antioxidant systems. Inhibition of *PGD* has been shown to deplete NADPH and trigger ferroptosis in cancer cells (27, 28), directly connecting *PGD* to ferroptosis regulation. In the context of sepsis, the overall activity of the pentose phosphate pathway, including glucose−6−phosphate dehydrogenase (G6PD), is altered during infection, and ferroptosis contributes to sepsis−associated acute kidney injury and lung injury (29). Therefore, the observed upregulation of *PGD* in neonatal sepsis likely represents a compensatory adaptive response to oxidative stress that simultaneously modulates cellular sensitivity to ferroptosis. Future studies are warranted to systematically evaluate the spatiotemporal expression changes of *PGD* in animal models of sepsis and to validate its impact on organ injury and survival through gene knockout or pharmacological inhibition. Collectively, these findings underscore the central role of oxidative stress-related cell death pathways in the progression of neonatal sepsis, consistent with previous reports of disrupted iron and copper metabolism in sepsis-induced organ injury (12, 14, 30).

Immune infiltration analysis using CIBERSORT revealed significant alterations in the immune landscape of neonatal sepsis patients, including elevated levels of neutrophils and regulatory T cells (Tregs), alongside decreased populations of CD8^+^ T cells and activated NK cells. This immunosuppressive phenotype resembles observations in adult sepsis, where excessive neutrophil activation and T cell exhaustion correlate with poor outcomes (31, 32). The strong correlations between *PGD*, *MAPK14*, and *KRAS* and specific immune cell subsets (e.g., neutrophils, CD8^+^ T cells) further highlight their potential regulatory roles in sepsis-associated immune dysregulation. Growing evidence indicates that ferroptosis and cuproptosis can modulate immune cell function and inflammatory responses (33, 34). Therefore, we hypothesize that these biomarkers may influence the fate and function of immune cells by regulating ferroptosis/cuproptosis pathways, thereby shaping the immune microenvironment in sepsis.

The translational value of this study lies in the systematic drug screening and molecular docking, which identified two FDA-approved tyrosine kinase inhibitors—dasatinib and gefitinib—as showing high-affinity binding (docking energy < -7 kcal/mol) to the *MAPK14* and *KRAS* proteins, respectively. This computational prediction was supported by *in vitro* experiments: both drugs significantly inhibited cell viability in an LPS-induced THP-1 macrophage model of sepsis.

This suggests an attractive drug repurposing strategy. Dasatinib, a multi-target kinase inhibitor, may, through its binding to *MAPK14*, inhibit the p38 MAPK signaling pathway, thereby attenuating downstream cytokine storms and cellular stress responses. The p38 MAPK pathway is known to regulate the expression and activity of ferroptosis-related proteins (e.g., ACSL4, GPX4); thus, inhibiting *MAPK14* might indirectly interfere with the ferroptosis process (20–22). Gefitinib, an EGFR inhibitor, may—through its interaction with *KRAS* (despite *KRAS* being a downstream signal transducer)—disrupt the complex RAS-MAPK signaling network, affecting cell proliferation, survival, and metabolic reprogramming (35, 36). *KRAS* mutation or activation has been shown to alter cellular redox status and metal ion metabolism, which may be linked to cuproptosis susceptibility (26). Consequently, these drugs might exert multi-faceted therapeutic effects in sepsis by directly or indirectly targeting *MAPK14* and *KRAS*, simultaneously modulating the axes of inflammation, ferroptosis, and cuproptosis.

While this study presents promising biomarkers and candidate drugs, certain limitations exist. Firstly, the retrospective nature and reliance on public transcriptomic data may introduce selection bias. Furthermore, although batch effects between the two validation platforms were addressed by ComBat and PCA confirmed the removal of platform−driven clustering, the possibility of subtle residual confounding cannot be entirely excluded. Future external validation in a large, single−platform, prospectively recruited cohort is warranted to definitively rule out any batch−related influences on the diagnostic estimates. Secondly, the lack of direct experimental validation in clinical samples or animal models limits the immediate translational impact of these findings. Notably, the LPS−stimulated THP−1 macrophage model used in this study reflects a simplified, monocyte/macrophage−driven inflammatory state and does not fully recapitulate the multifaceted pathophysiology of neonatal sepsis; therefore, it served solely to confirm the expression trends of the candidate biomarkers under inflammatory conditions, rather than to faithfully mimic the disease. In addition, a practical concern lies in the turnaround time of RT−qPCR, which, at several hours, may not outperform some existing rapid diagnostic protocols. However, we contend that the principal value of this work lies in the specificity of the molecular signature, which could improve diagnostic accuracy compared with non−specific clinical signs and conventional biomarkers such as CRP. Emerging point-of-care nucleic acid testing technologies are expected to overcome the obstacle of testing turnaround time in the future, thereby accelerating the application of such molecular assays. Therefore, the emphasis of this biomarker panel shifts from speed alone to its diagnostic accuracy and biological rationale. Future research should validate these biomarkers in prospective cohorts and utilize *in vitro* and *in vivo* sepsis models to deeply explore their mechanistic roles.

## 5 Conclusion

This study identifies *PGD*, *MAPK14*, and *KRAS* as diagnostic biomarkers for neonatal sepsis and reveals their association with ferroptosis, cuproptosis, and immune dysregulation. By integrating machine learning, immune infiltration analysis, and molecular docking, we established a general framework for biomarker discovery and screened candidate drugs—dasatinib, gefitinib, sorafenib and vandetanib—that showed binding affinity to the target proteins. These findings suggest the possible value of targeting oxidative stress-related cell death pathways and their upstream regulatory nodes (e.g., *MAPK14*, *KRAS*) for early diagnosis and therapy in neonatal sepsis. Further experimental validation and clinical studies are needed to assess whether these computational predictions can be translated into clinical practice.

## Data Availability

The original contributions presented in the study are included in the article/**Supplementary Material**. Further inquiries can be directed to the corresponding author.
