# Supplementary material for: Computational discovery of PGD, MAPK14, and KRAS as diagnostic biomarkers for neonatal sepsis through integrated machine learning, immune infiltration analysis, and molecular docking
Source: Front Immunol. 2026 May 29;17:1808072. doi: 10.3389/fimmu.2026.1808072 (PMC13259686; doi:10.3389/fimmu.2026.1808072)
Supplement: Supplementary file 1 [file DataSheet1.docx]

Table S1. Information on datasets obtained from GEO

| GEO dataset | Platform | Neonatal sepsis | Normal |
| --- | --- | --- | --- |
| GSE69686 | GPL20292 | 64 | 85 |
| GSE25504 | GPL6947 | 26 | 37 |
|  | GPL13667 | 14 | 6 |

Table S2. The clinical characteristics of the patients in the validation group

| Characteristics | Neonatal sepsis | Normal |
| --- | --- | --- |
| Gender | Female 16; Male 24 | Female 14; Male 29 |
| Age (Day) | 226 (178-279) | 267 (206-309) |
| Birthweight (g) | 1167 (450-3900) | 3563 (670-4590) |


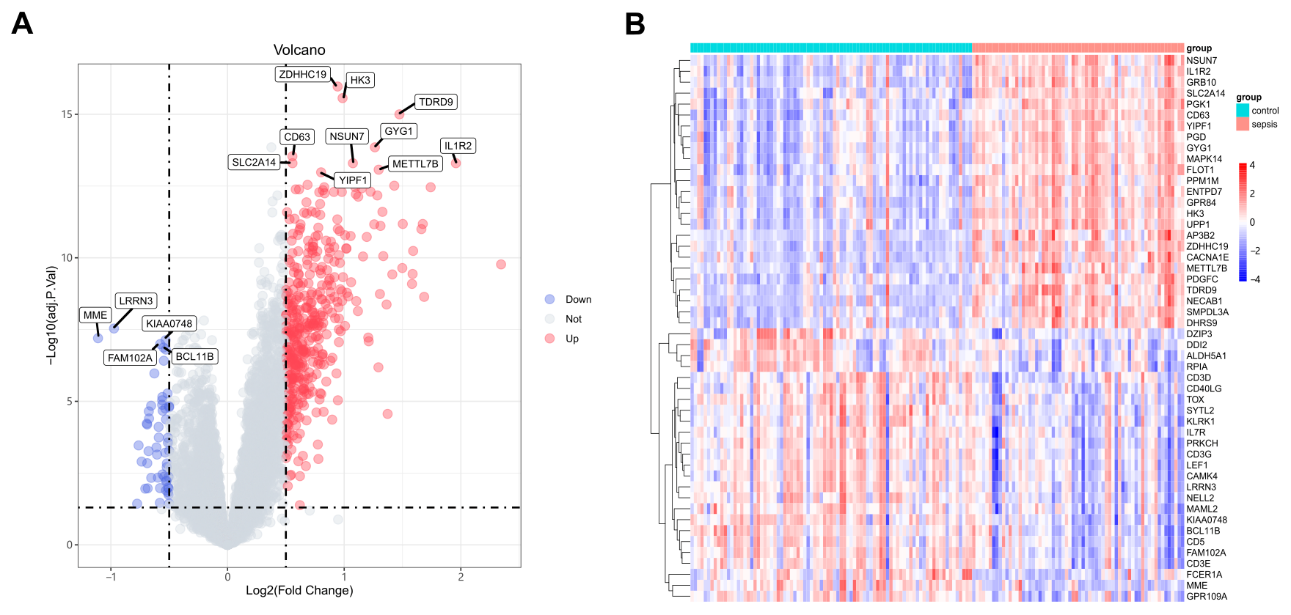


**Fig. S1** Identification of DEGs associated with neonatal sepsis. (A) Volcano plot of DEGs in patients with neonatal sepsis and normal controls; (B) Heatmap of DEGs in patients with neonatal sepsis and normal controls.


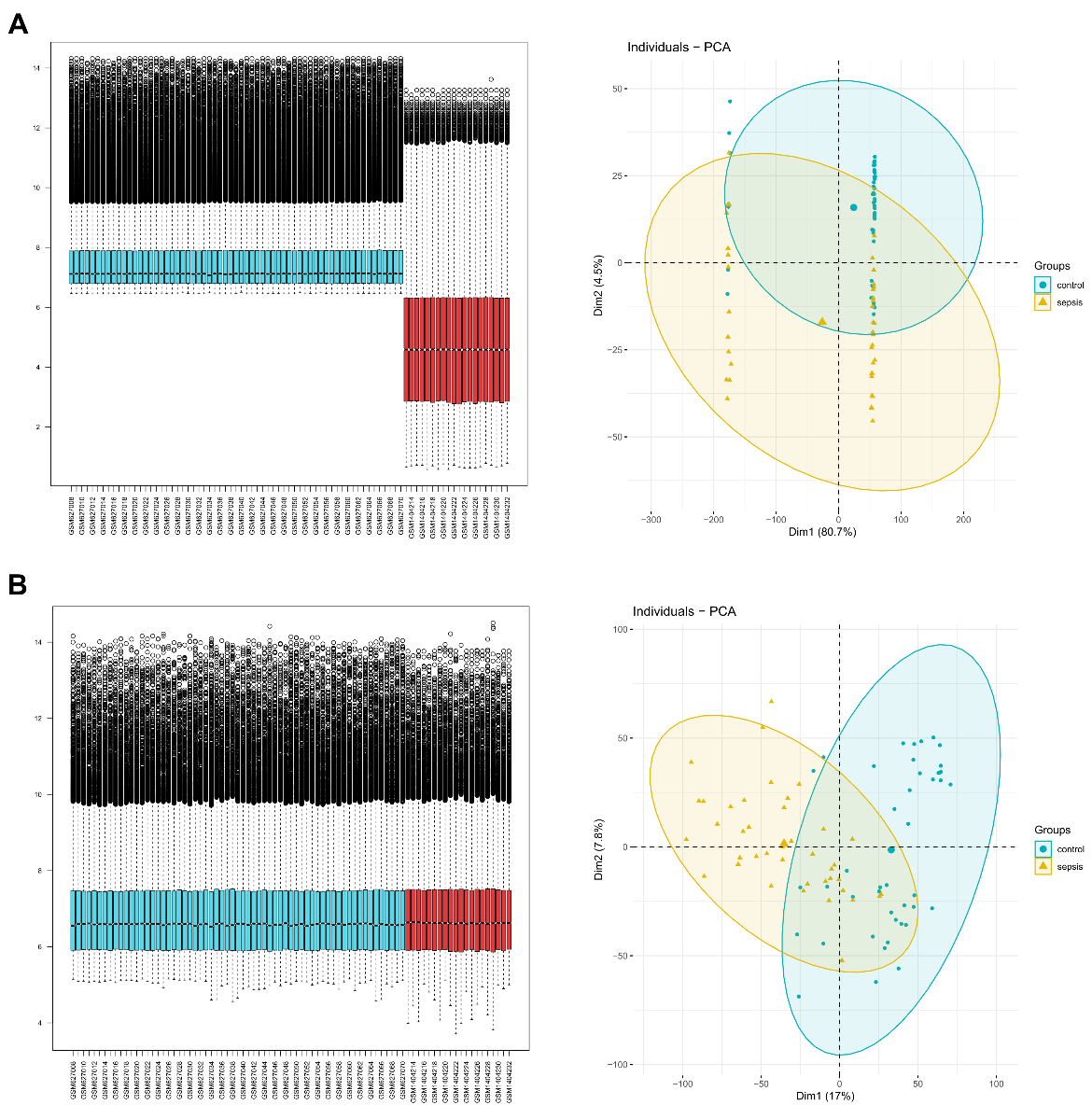


**Fig. S2** Processing validation set data. (A) Boxplots and Principal component analysis (PCA) plots of the validation set before batch effects were removed; (B) Boxplots and PCA plots of the validation set after removing the batch effect.
